# Application of Internet Hospitals in the Disease Management of Patients With Ulcerative Colitis: Retrospective Study

**DOI:** 10.2196/60019

**Published:** 2025-03-18

**Authors:** Tianzhi Yu, Wanyu Li, Yingchun Liu, Chunjie Jin, Zimin Wang, Hailong Cao

**Affiliations:** 1 Internet Hospital Tianjin Medical University General Hospital Tian Jin China; 2 Department of Gastroenterology National Key Clinical Specialty Tianjin Medical University General Hospital Tianjin China; 3 School of Management Tianjin University of Traditional Chinese Medicine Tian Jin China

**Keywords:** inflammatory bowel disease, ulcerative colitis, intelligent diagnosis and treatment service, internet hospital, chronic disease management

## Abstract

**Background:**

Ulcerative colitis (UC) is a chronic disease characterized by frequent relapses, requiring long-term management and consuming substantial medical and social resources. Effective management of UC remains challenging due to the need for sustainable remission strategies, continuity of care, and access to medical services. Intelligent diagnosis refers to the use of artificial intelligence–driven algorithms to analyze patient-reported symptoms, generate diagnostic probabilities, and provide treatment recommendations through interactive tools. This approach could potentially function as a method for UC management.

**Objective:**

This study aimed to analyze the diagnosis and treatment data of UC from both physical hospitals and internet hospitals, highlighting the potential benefits of the intelligent diagnosis and treatment service model offered by internet hospitals.

**Methods:**

We collected data on the visits of patients with UC to the Department of Gastroenterology at Tianjin Medical University General Hospital. A total of 852 patients with UC were included between July 1, 2020, and June 31, 2023. Statistical methods, including chi-square tests for categorical variables, *t* tests for continuous variables, and rank-sum tests for visit numbers, were used to evaluate the medical preferences and expenses of patients with UC.

**Results:**

We found that internet hospitals and physical hospitals presented different medical service models due to the different distribution of medical needs and patient groups. Patients who chose internet hospitals focused on disease consultation and prescription medication (3295/3528, 93.40%). Patients’ medical preferences gradually shifted to web-based services provided by internet hospitals. Over time, 58.57% (270/461) of patients chose either web-based services or a combination of web-based and offline services for UC diagnosis and treatment. The number of visits in the combination of web-based and offline service modes was the highest (mean 13.83, SD 11.07), and younger patients were inclined to visit internet hospitals (49.66%>34.71%). In addition, compared with physical hospitals, there was no difference in testing fees and examination fees for patients with UC in internet hospitals, but medicine fees were lower.

**Conclusions:**

The intelligent diagnosis and treatment model provided by internet hospitals demonstrates the potential benefits in managing UC, including feasibility, accessibility, convenience, and economics.

## Introduction

Ulcerative colitis (UC) is a chronic inflammatory bowel disease (IBD) affecting the rectum and colon, characterized by a relapsing and remitting course. As of 2023, UC affects an estimated 5 million individuals globally, with a rapidly increasing incidence [1]. This growing prevalence imposes a substantial economic burden on both health care systems and patients.

Uncontrolled UC can lead to severe complications, including malnutrition [2], intestinal infections [3,4], colon cancer [5], anemia, perforation, toxic megacolon [6], and sepsis. These complications not only decrease the quality of life but also increase the risk of mortality. Effective management strategies are essential to reduce these risks.

Traditionally, patients with UC have required frequent clinic visits and hospitalizations due to the complexity of the disease. To overcome these challenges, several telehealth models have been developed to monitor disease activity and facilitate management, reducing the number of outpatient visits and hospital admissions while improving medication adherence [7,8]. Recent advancements in artificial intelligence (AI), particularly large language models such as ChatGPT, show promise in supporting chronic disease management [9,10]. Studies have highlighted their potential for interpreting medical information and generating responses to common patient queries, thereby enhancing patient education and engagement. Despite these advancements, limitations remain, including short follow-up periods, restricted generalizability to broader UC populations, lack of common-sense judgment, and delays in information updates. In addition, existing telehealth models often rely heavily on pharmacists for medication management [11-13], but greater clinician involvement is essential to ensure comprehensive care for patients with UC.

The internet hospital, as a digital expansion of traditional medical services, uses advanced information and communication technologies to offer consultation, prescription, and follow-up services [14]. Internet hospitals established by physical hospitals can connect web-based and offline medical services, ensuring the continuity of care and adequately meeting the medical needs of patients. The COVID-19 pandemic significantly accelerated their development, as government initiatives promoted the use of internet-based health care platforms to enhance access and continuity of care [15-17]. By early 2023, over 3000 internet hospitals were operating across China, demonstrating the growing importance of this model in managing chronic diseases such as UC. Since internet hospitals are not constrained by geographical limitations, patients can conveniently download the client software for internet hospital services on their mobile phones and schedule appointments for various medical services. Consequently, internet hospitals enhance the accessibility and convenience of health care delivery, particularly in addressing the challenges faced by chronic patients who frequently visit hospitals and experience prolonged waiting times for medical treatment.

Based on the current situation and the development trends in health care, we propose 4 hypotheses regarding internet hospitals in the context of UC management. First, internet hospitals can comprehensively meet the medical service needs of patients with UC by leveraging intelligent tools for diagnosis, treatment, and follow-up. Second, more patients with UC will opt for internet hospital services in the future, drawn by the convenience of digital health care. Third, the accessibility of internet hospitals will lead to an increase in the frequency of visits by patients with UC. Finally, internet hospital services are more economical, reducing costs such as transportation and medicine fees.

## Methods

### Data Sources

**Figure 1:**
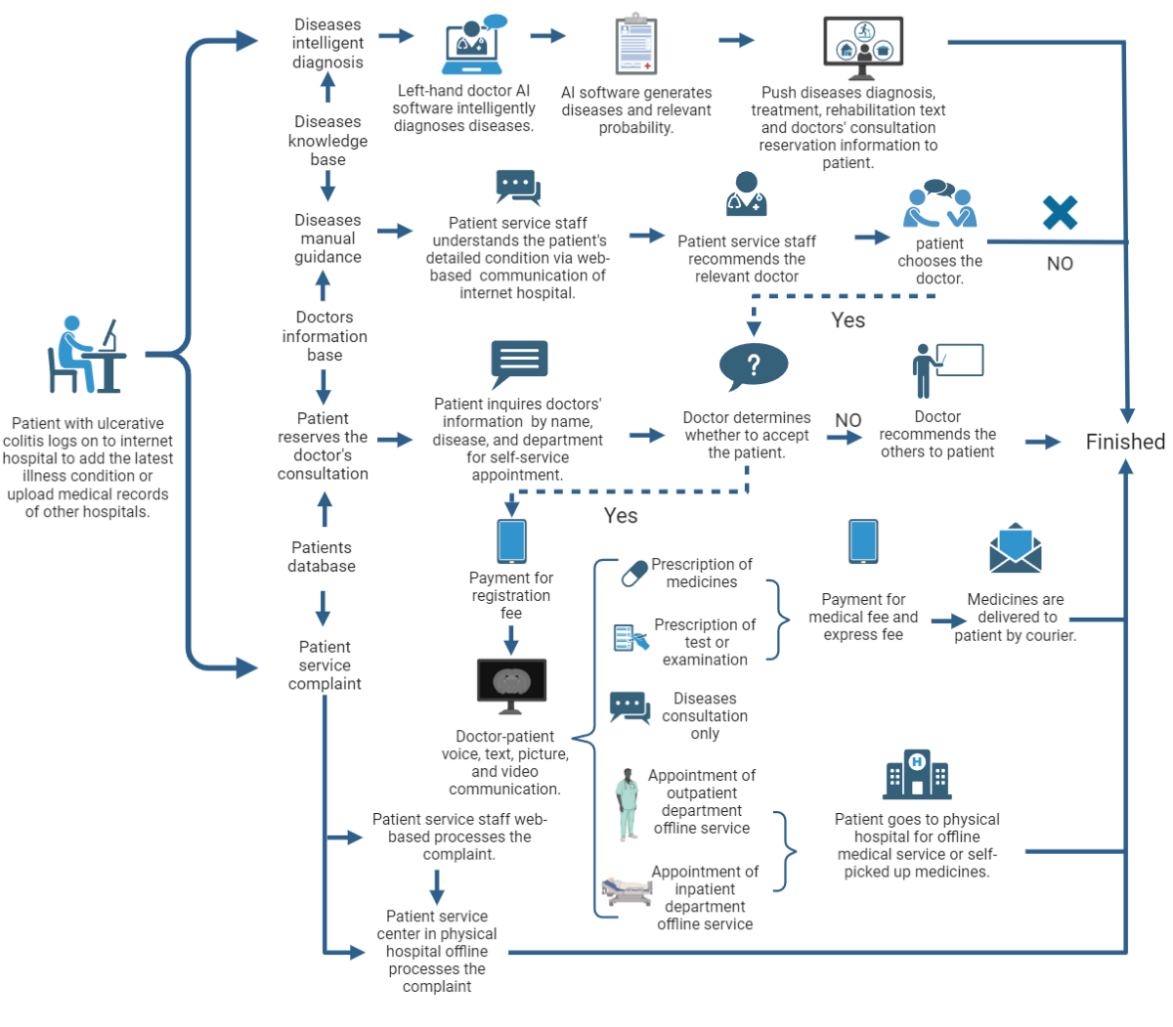
The flowchart of the internet hospital’s web-based service process. AI: artificial intelligence.

The data for this study were obtained from Tianjin Medical University General Hospital, a Grade-A tertiary general hospital located in Tianjin. In response to the COVID-19 epidemic and the need for effective prevention and control measures, Tianjin Medical University General Hospital established an internet hospital, which officially commenced operations in March 2020. The intelligent diagnosis system in the internet hospital uses AI-based tools, such as the Left-Hand Doctor software, as an auxiliary diagnosis and treatment tool., This system uses deep learning algorithms and big data processing to evaluate symptoms and generate diagnostic probabilities. It also integrates interactive dialogue systems to guide patients through their treatment journey [18,19]. The flowchart of the internet hospital’s web-based service is shown in Figure 1.

### Data Collection and Inclusion Criteria

**Figure 2:**
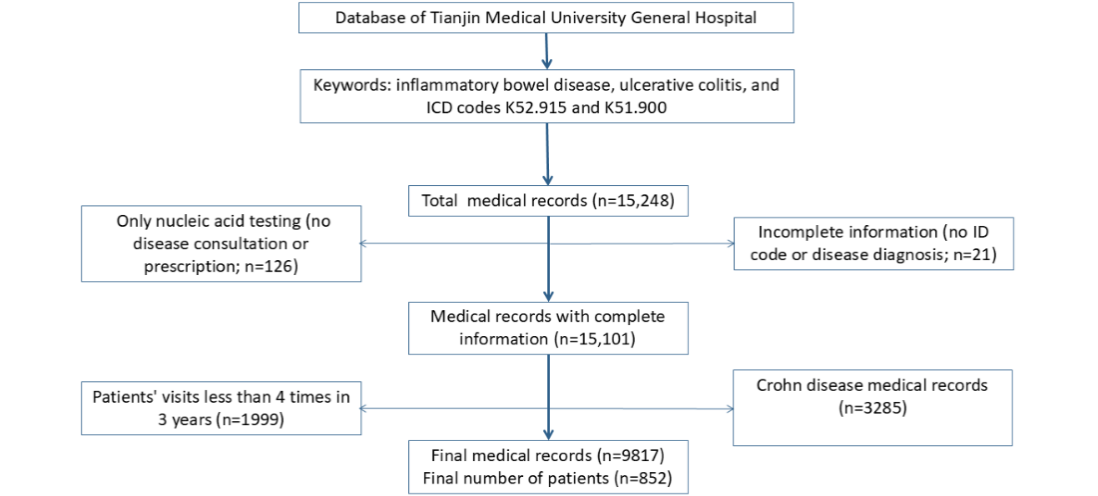
Data collection flowchart. ICD: International Classification of Diseases.

We retrieved 15,248 medical records using keywords related to IBD and UC, including *ICD* (*International Classification of Diseases*) codes K52.915 and K51.900, from the database of Tianjin Medical University General Hospital. After excluding 21 incomplete records, 1999 cases with no more than 4 consultations were excluded, as UC is a chronic disease requiring regular follow-up. We believe that if patients visit fewer than 4 times in 3 years, their data may be incomplete, and new patients may not have developed medical habits. In addition, infrequent visits do not accurately reflect preferences for offline or web-based services. We also excluded 126 cases involving only COVID-19 nucleic acid testing and 3285 Crohn disease records. Ultimately, 852 patients were included, with a total of 9817 UC-related visits over 3 years. Of these, 445 (52.2%) were male patients, 407 (47.8%) were female patients, and 649 (76.2%) patients had basic health insurance. This data collection flowchart is shown in Figure 2.

### Study Design

In this study, we extracted the diagnosis and treatment data of patients with UC from the Department of Gastroenterology between July 1, 2020, and June 31, 2023. The dataset includes data from in-person consultations at the physical hospital outpatient department and web-based consultations through the internet hospital over the same period. The data also includes information about patients with UC, such as their gender, age, disease diagnosis, treatment time, treatment method, medical expenses, and medical insurance. Our research team tracked the associated patients’ visits to UC over 3 years, using their identification card numbers as unique identifiers. Starting in July 2020, the data were divided into 6-month periods to discover the changing trends in the medical services sought by patients with UC, as the promotion and application of internet hospitals continued to grow.

### Measures

Medical preferences refer to the types of medical modalities that patients with UC select based on their individual needs, experiences, and the advantages of each modality. From an empirical perspective, we analyze the number of patients using different medical service modes over time to illustrate trends.

The economy of internet hospitals specifically pertains to the comparison of web-based medical fees with those charged in offline settings, including testing fees, examination fees, and medicine fees. Furthermore, patients who opt for internet hospital services can save time and transportation costs for themselves and their companions.

### Statistical Analysis

We used chi-square tests to analyze differences in medical preferences and service demands between patients with UC using physical or internet hospitals. We used *t* tests to compare medical expenses across the 2 service modes, while rank-sum tests were used to evaluate differences in the number of patient visits among different medical modes. Frequency and percentages were used to characterize noncontinuous variables, including gender, age group, patient needs, and the number of patients. In contrast, mean and SD values were used to describe continuous variables such as the number of visits and medical fees. All data analysis was performed using IBM SPSS Statistics (version 25).

### Ethical Considerations

This study was reviewed and approved by the Ethics Committee of the Tianjin Medical University General Hospital (approval number IRB2024-YX-066-01). The study involved analyzing patient data from the hospital database and did not involve human experimentation or compensation. The data used for this research were extracted from the hospital’s information system with previous approval from the Tianjin Medical University General Hospital. To ensure patient privacy and confidentiality, identifying information such as names, addresses, and contact details were removed during data extraction. As a result, the datasets used in the study were anonymized, and no personally identifiable information was accessible to the research team. All patient-related datasets met ethical and legal requirements for data protection. The study adhered to the principles of informed consent, and participants were fully informed of the study’s purpose and procedures. In addition, the research team complied with all relevant local, national, and international laws and regulations regarding the protection of personal information, privacy, and human rights. The Ethics Committee of the Tianjin Medical University General Hospital granted approval for this study following a thorough review of the research protocol.

## Results

### Characteristics of Web-Based and Offline Service Demand

The web-based service mode of the internet hospital can provide equal diagnosis and treatment as a physical hospital. However, web-based consultations (n=3528) were fewer than offline consultations (n=6289), with a significant difference in service demands (*P*<.001, Table 1). The primary web-based service was prescribing medication (2071/3528, 58.70%), followed by disease consultations (1224/3528, 34.70%). These findings indicated that web-based services played an important role in the diagnosis and treatment needs of patients with UC, meeting their basic needs, and facilitating follow-up and disease management for chronic diseases. These findings highlight the role of web-based platforms in addressing the basic needs of patients with UC and supporting follow-up care and disease management.

**Table 1:** Patients’ service needs and numbers of visits across different age cohorts over 3 years (N=9817; χ23=879.9, *P*<.001).

Patients’ demands for diagnosis and treatment services	Web-based service, n/N (%)	Offline service, n/N (%)
Prescribe medicine	2071/3528 (58.70)	4634/6289 (73.69)
Prescribe testing and examination	125/3528 (3.54)	553/6289 (8.79)
Prescribe drugs, testing, and examination	108/3528 (3.06)	419/6289 (6.66)
Disease consultation only	1224/3528 (34.70)	683/6289 (10.86)

### Preference of Different Ages for Medical Treatment Modes

Patients with UC in the age groups of younger than 30 years and between 30 and 40 years demonstrated a greater preference for web-based consultations, with rates of 49.66% and 34.71%, respectively. In contrast, patients aged 50 years and older predominantly opted for in-person consultations at physical hospitals, as indicated by their preference rates of 43.09% compared with 28.03% in Table 2.

**Table 2:** Patients’ visit numbers for different age cohorts across different diagnosis and service modes over 3 years (N=9817; χ24=278.7, *P*<.001).

Patients’ visit numbers of different age cohorts	Web-based service, n/N (%)	Offline service, n/N (%)
Age≤30 years	621/3528 (17.60)	712/6289 (11.32)
30<Age≤40 years	1131/3528 (32.06)	1471/6289 (23.39)
40<Age≤50 years	787/3528 (22.31)	1396/6289 (22.20)
50<Age≤60 years	493/3528 (13.97)	1156/6289 (18.38)
Age>60 years	496/3528 (14.06)	1554/6289 (24.71)

### Changing Trend of Diagnosis and Treatment Service Modes

**Figure 3:**
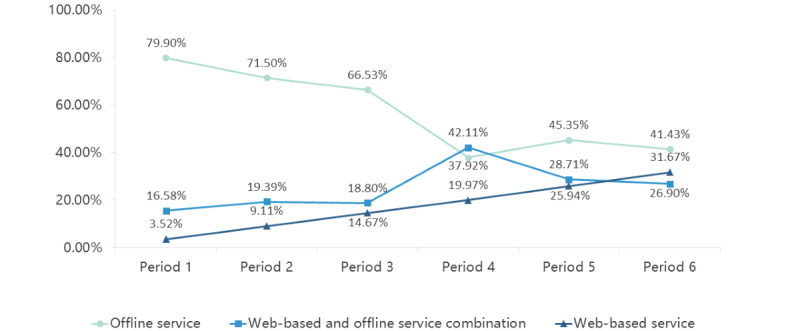
Percentage of patients choosing different diagnosis and treatment service modes across 6 periods.

During period 1, only 3.52% of patients opted for completely web-based services, while the majority (n=318, 79.90%) preferred entirely offline services, as given in Table 3. By period 6, there was a significant increase in the percentage of patients choosing either completely web-based services (n=146, 31.67%) or a combination of web-based and offline services (n=124, 26.90%), together comprising a total of 58.57%. In contrast, the proportion of patients selecting exclusively offline services decreased from 79.90% in Period 1 to just 41.43% by period 6. The trend of patients selecting various diagnosis and treatment service modes over the 6 periods is given in Figure 3. This trend reflects the growing acceptance and use of web-based diagnosis and treatment options among patients with UC.

**Table 3:** Number (percentage, %) of patients choosing different diagnosis and treatment service modes across 6 periods (χ210=368.9, *P*<.001).

Periods	Offline service, n (%)	Web-based and offline service combination, n (%)	Web-based service, n (%)
Period 1	318 (79.90)	66 (16.58)	14 (3.52)
Period 2	306 (71.50)	83 (19.39)	39 (9.11)
Period 3	322 (66.53)	91 (18.80)	71 (14.67)
Period 4	262 (37.92)	291 (42.11)	138 (19.97)
Period 5	229 (45.35)	145 (28.71)	131 (25.94)
Period 6	191 (41.43)	124 (26.90)	146 (31.67)

### Preference of Medical Treatment Modes

The number of patients (n=498, 58.45%) who opted for a combination of web-based and offline services, as well as completely web-based consultations, exceeded that of those choosing physical hospitals (354/852, 41.55%) in Table 4. However, patients who chose the combination of web-based and offline care had the highest number of visits (mean 13.83, SD 11.07).

**Table 4:** Number of patient visits for consultations with different medical service modes over 3 years (N=852; Kruskal-Wallis H=51.39, *P*<.001).

Service modes	Number of patients, n (%)	Number of visits, mean (SD)
Offline service	354 (41.55)	9.33 (6.78)
Web-based and offline service combination	424 (49.77)	13.83 (11.07)
Web-based service	74 (8.68)	8.80 (6.81)

### Difference in the Fee of Web-Based and Offline Treatment

The testing fees and examination fees for patients with UC in different age groups showed no statistical difference. However, there was a significant disparity in medicine fees for web-based and offline services among patients with UC of all age groups, with web-based drug fees being lower than offline ones, as given in Table 5.

**Table 5:** Web-based and offline service expenses (yuan) for different age cohorts (N=9817).

Service expenses		Age≤30 years	30<Age≤40 years	40<Age≤50 years	50<Age≤60 years	Age>60 years
**Testing fee**						
	Web-based service, mean (SD)	289.19 (24.79)	293.49 (23.74)	288.75 (25.32)	325.23 (41.22)	264.90 (46.69)
	Offline service, mean (SD)	322.26 (29.99)	325.18 (23.76)	307.42 (37.13)	328.38 (25.38)	266.10 (19.10)
	T-value	0.92	0.94	0.43	0.06	0.06
	*P* value	.36	.35	.67	.95	.98
**Examination fee**						
	Web-based service, mean (SD)	300.00 (13.63)	393.66 (38.22)	381.03 (40.41)	364.44 (19.17)	413.44 (42.49)
	Offline service, mean (SD)	342.14 (11.73)	391.58 (13.30)	404.55 (15.87)	385.99 (16.20)	384.38 (13.48)
	T-value	1.81	0.06	0.60	0.57	0.62
	*P* value	.72	.95	.55	.57	.53
**Medicine fee**						
	Web-based service, mean (SD)	731.65 (24.41)	807.03 (14.65)	747.66 (19.40)	700.99 (23.34)	823.04 (23.95)
	Offline service, mean (SD)	922.16 (27.14)	996.65 (17.25)	879.55 (15.79)	912.57 (17.65)	914.70 (14.74)
	T-value	5.22	8.38	5.27	7.23	3.26
	*P* value	<.001	<.001	<.001	<.001	<.001

## Discussion

### Principal Findings

The findings indicate that internet hospitals can effectively address the needs of patients with UC. An increasing number of patients with UC, particularly younger individuals, are opting for internet hospitals for their treatment and follow-up care. This trend not only facilitates better management of UC but also contributes to a reduction in health care costs associated with the disease.

The Tianjin Medical University internet hospital platform features the Left Hand Doctor AI software, which provides relevant information on disease diagnosis, treatment, rehabilitation, and available specialists. Consequently, the AI-driven model enhances patients’ ability to choose the appropriate clinical departments and health care providers. In contrast to certain internet hospitals that lack support from physical facilities, medications prescribed by this platform can be obtained either through self-pickup at a physical location or via express delivery. In addition, examination and testing procedures can be conducted at a physical hospital. Patients unable to receive a diagnosis through web-based consultations may be referred to a physical hospital’s outpatient department for further treatment. Those needing inpatient care can schedule hospitalization appointments directly via the internet hospital platform. In addition, medical disputes can be mediated at the reception service center of the physical hospital. This indicates that the internet hospital effectively facilitates continuity between web-based and offline medical services.

The COVID-19 pandemic has significantly accelerated the adoption of internet hospitals, with video consultations increasingly replacing many in-person visits [20]. Our findings demonstrate that web-based platforms, such as internet hospitals, play a pivotal role in addressing the long-term management needs of patients with UC. The majority of patients using web-based services sought medication prescriptions (58.70%) and disease consultations (34.70%), indicating the practicality of these platforms for chronic disease management and effectively reducing the burden on offline medical resources. Furthermore, our discoveries reveal a significant increase over time in the number of patients choosing fully web-based services or a combination of web-based and offline services. This trend suggests that internet hospitals will become increasingly accessible in the future. Together, these findings highlight the value of integrating web-based and offline services to ensure comprehensive care, especially in resource-constrained settings.

Age-based preferences indicated that younger patients (younger than 40 years) were more likely to use web-based consultations (49.66%), while older patients (50 years and older) predominantly chose offline services (43.09%). This suggests that emerging digital health methods, such as internet hospitals, are more easily accepted by younger people. The flexibility and convenience of web-based platforms appeal to younger demographics, whereas older patients may favor the familiarity and perceived reliability of in-person care. These trends highlight the need for a hybrid model to accommodate diverse patient preferences. Furthermore, web-based services significantly reduced medicine costs compared with offline services (*P*<.001), with consultation fees also being lower under the unified internet hospital policy in Tianjin. These cost advantages make web-based platforms a more economical option for the long-term management of UC, alleviating the financial burden on patients.

Our findings align with previous studies that demonstrated the growing role of telemedicine in managing chronic diseases. For example, de Jong et al [21] showed that telemedicine systems, such as myIBDcoach, significantly reduced hospital admissions and improved medication adherence in patients with IBD [21]. Similarly, 2 other studies highlighted the effectiveness of telehealth models in improving patient outcomes [22,23]. While these studies focused on specific telehealth tools, our research provides a broader analysis of internet hospital services, demonstrating their capacity to integrate web-based and offline care while maintaining cost-effectiveness and accessibility. However, unlike earlier studies that relied on short follow-up periods [24], our analysis spans 3 years, offering a more comprehensive view of the long-term impact of internet hospitals. A recent study by Al-Sheikh et al [25] emphasized the efficacy of telehealth models in reducing health care costs in IBD management [25]. However, it was unable to eliminate the cost variance resulting from the disparity in hospital registration techniques, as the research participants were from different hospitals. The participants of our study are all from the General Hospital of Tianjin Medical University, thereby effectively avoiding the interference of hospital technical factors.

### Strengths and Limitations

Our study has several strengths. First, this retrospective study provides insights into the use of internet and physical hospitals for UC management over 3 years, with a representative sample from a Grade-A tertiary hospital. Second, it is the first to investigate how internet hospitals can facilitate standardized care for patients with UC outside traditional settings. However, this study has limitations. The internet hospital system lacks data on the quality of care and patient satisfaction, which limits the evaluation of service outcomes. A service quality model, a satisfaction degree questionnaire, and even a comprehensive satisfaction degree index measured by a 5-point Likert scale are proposed to be designed based on the conditions of the internet hospitals and then integrated into the hospital information system. Therefore, quality and satisfaction data can be automatically obtained from the hospital database. Furthermore, long-term studies are needed to assess the sustained effects of internet hospitals on patient outcomes, as well as the accuracy and precision of AI diagnosis tools such as the Left-Hand Doctor mentioned in the paper.

### Conclusion

Internet hospitals represent an innovative approach to UC management, offering a series of web-based intelligent diagnosis and treatment services. Through seamless integration of web-based and offline care, they ensure continuity of care. Compared with the traditional medical model, this approach highlights the potential benefits in terms of feasibility, accessibility, convenience, and economy. This intelligent mode can also be applied to the medical service and disease management of other chronic diseases.

## Appendix

Multimedia Appendix 1 The full list of keywords and search strategy of the database.

## Abbreviations

AI: artificial intelligence
IBD: inflammatory bowel disease
ICD: International Classification of Diseases
UC: ulcerative colitis

## Data Availability

The datasets generated or analyzed during this study are available from the corresponding author on reasonable request.
