# Supplementary material for: Application of Internet Hospitals in the Disease Management of Patients With Ulcerative Colitis: Retrospective Study
Source: J Med Internet Res. 2025 Mar 18;27:e60019. doi: 10.2196/60019 (PMC11962335; doi:10.2196/60019)
Supplement: Multimedia Appendix 1 [file jmir_v27i1e60019_app1.docx]

Retrieval strategy

The research team and information engineers collaboratively developed a retrieval strategy. Medical information of Tianjin Medical University General Hospital is stored in electronic medical records (EMR) and the hospital information system (HIS) database, both of which are SQL Server databases. Based on the International Classification of Diseases (ICD) codes K52.915 and K51.900, we performed data retrieval in both the EMR and HIS databases using SQL queries. The entire retrieval process utilized the patient’s Identity document (ID) card number as a unique identifier to link patients’ basic demographic information, outpatient department offline service data, and internet hospital online service data. This approach effectively ensured the accuracy, authenticity, and completeness of the retrieved data. The key words extracted include patients’ ICD code, Registration Number, Gender, Age, ID number, Registration number, Clinical department code, Cinical department name, Medcial service mode ( Online or Offline), Medical insurance, Test items, Test fee, Examination items, Examination fee, Medicine, Medicine fee, Diagnosis.

At last 15248 medical records are obtained, After excluding 21 incomplete records, 1,999 cases with no more than four consultations（UC is a chronic disease requiring regular follow-up. We believe that if patients visit fewer than four times in three years, their data may be incomplete, and new patients may not develop medical habits. Additionally, infrequent visits do not accurately reflect preferences for offline or online services）, 126 cases involving only COVID-19 nucleic acid testing, and 3,285 Crohn's disease records, we included 852 patients with a total of 9,817 UC-related visits over three years. There were 445 (52.2%) male patients and 407 (47.8%) female patients included in this study, and 649 (76.2%) patients with basic health insurance.

Key words

ICD code, Registration Number, Gender, Age, ID number, Registration number, Clinical department code, Cinical department name, Medcial service mode (Online or Offline), Medical insurance, Test items, Test fee, Examination items, Examination fee, Medicine, Medicine fee, Diagnosis
